# The Energy Costs of Prematurity and the Neonatal Intensive Care Unit (NICU) Experience

**DOI:** 10.3390/antiox7030037

**Published:** 2018-03-02

**Authors:** John B. C. Tan, Danilo S. Boskovic, Danilyn M. Angeles

**Affiliations:** 1 Division of Biochemistry, Department of Basic Sciences, Loma Linda University, Loma Linda, CA 92350, USA; jctan@llu.edu (J.B.C.T.); dboskovic@llu.edu (D.S.B.); 2 Division of Physiology, Department of Basic Sciences, Loma Linda University, Loma Linda, CA 92350, USA

**Keywords:** premature neonate, energy, nutrition, pain, sucrose

## Abstract

Premature neonates are in an energy deficient state due to (1) oxygen desaturation and hypoxia events, (2) painful and stressful stimuli, (3) illness, and (4) neurodevelopmental energy requirements. Failure to correct energy deficiency in premature infants may lead to adverse effects such as neurodevelopmental delay and negative long-term metabolic and cardiovascular outcomes. The effects of energy dysregulation and the challenges that clinicians in the Neonatal Intensive Care Unit (NICU) face in meeting the premature infant’s metabolic demands are discussed. Specifically, the focus is on the effects of pain and stress on energy homeostasis. Energy deficiency is a complex problem and requires a multi-faceted solution to promote optimum development of premature infants.

## 1. Introduction

Premature infants (less than 37 weeks gestational age) face numerous challenges during their stay in the neonatal intensive care unit (NICU). Not only do they face the challenge of being underdeveloped compared to term infants, they are also at risk to a variety of illnesses, as well as undernutrition and growth failure [1,2], These factors lead to a state of energy deficiency and catabolism, with potential long-term effects such as impaired neuronal development [3,4] and metabolic diseases [5,6]. Critically ill neonates and premature infants in particular, also undergo multiple (from 4 to 16) tissue damaging procedures (TDPs) for clinical care or diagnostic purposes [7,8,9]. These procedures include tape removal, heel-stick, venipuncture, intravenous or central catheter placement, injections and tracheal suctioning and intubation. Analgesic treatments for TDPs usually come in the form of 0.5 mL to 2 mL/kg per dose of a 24% oral sucrose solution given with a pacifier, lingually or at the buccal mucosa [10,11,12,13]. However, sucrose may play an indirect role in the modulation of the premature infant’s stress physiology, metabolism and energy state [10,14,15,16,17,18]. With this in mind, the premature condition in the context of routine clinical procedures, energy metabolism and prospective long-term outcomes of energy deficit are of interest. The administration of oral sucrose and its potential effects on energy metabolism and stress physiology require particular attention. It is anticipated that, with improved understanding of the potential mechanisms of the development of energy deficit in premature neonates, rational preventive and treatment approaches will emerge. The objective of this narrative review is to summarize the complexity of the metabolic demands of the premature neonate, as well as the potential consequences of not meeting these demands, leading to energy deficit.

## 2. Premature Infants Are in an Energy-Deficit State

**Figure 1:**
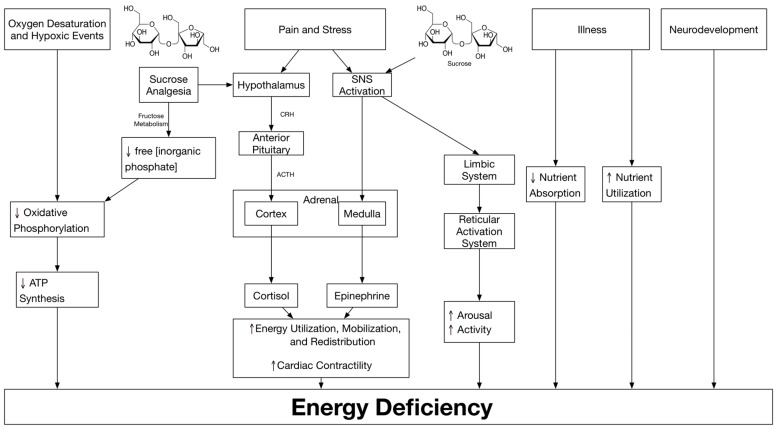
Causes of energy deficiency in the premature infant. (ATP, adenosine triphosphate; SNS, sympathetic nervous system).

Preterm births steadily increased from 9.5% in 1981 to 12.7% in 2005 [19]. During the first few weeks of postnatal life, premature infants are most likely ill, physiologically unstable and lack adequate nutritional support [2]. Following birth, these infants simultaneously experience a loss of maternal nutritional support along with their own limited ability for energy storage [20,21]. Energy deficit occurs due to four possible reasons (see Figure 1 for a simplified diagram).

First, there may be reduced energy stores due to oxygen desaturation events that occur frequently in the NICU setting, most often due to prematurity or respiratory disease [22]. Neonates experiencing multiple oxygen desaturation events are at risk for hypoxia, which refers to an inadequate oxygen supply to the tissues. This leads to a reduced rate of oxidative phosphorylation by aerobic respiration and to reduced adenosine triphosphate (ATP) synthesis [23]. Because of the lack of oxygen as a final electron acceptor, the mitochondria are unable to maintain the proton gradient necessary to form ATP from adenosine diphosphate (ADP) and inorganic phosphate [24]. Consequently, ATP production is reduced and possibly interrupted.

Second, energy stores may be reduced in response to stressful stimuli such as procedural pain. We explored the effects of tissue damaging procedures (TDPs) on ATP metabolism [25]. When the TDP was tape removal, performed along with the removal of a central or venous catheter, we found a significant increase in uric acid (UA) and malondialdehyde (MDA) thirty minutes after the painful stimulus. UA is a downstream product of ATP degradation. MDA is an oxidative stress marker formed by the oxidative degradation of polyunsaturated lipids by reactive oxygen species (ROS). The increase in ATP degradation in response to painful procedures may be due to energy expended through behavioral and physiological reactions to pain, such as crying, facial grimacing, flailing and tachycardia [26]. The increased oxidative stress could be due to increased purine degradation with concomitant production of ROS. Alternatively, oxidative stress could also be due to increased mitochondrial ATP synthesis activity, of which ROS is a byproduct, in order to meet the energy demands of increased ATP utilization [27]. Both the increased ATP utilization and oxidative stress can lead to energy deficit.

Third, energy stores can be reduced during illness [2]. Though current understanding for the neonatal population is modest, studies in adult and children show that critical illness changes metabolism significantly by decreasing the rate of absorption and utilization of nutrients [28,29,30,31]. Furthermore, elevated pro-inflammatory cytokines, such as TNF-α, IL-6 and IL-10 play a role in increasing metabolic demand [29,32]. Such pro-inflammatory cytokines were found to be elevated in critically ill neonates, implying that increased metabolic demand can also occur in the premature neonate population [32,33]. An increase in metabolic demand will further decrease energy stores.

Finally, an increase in metabolic demand due to the high energy demands of the brain [34,35,36] can further reduce a premature neonate’s energy stores. The neonatal brain accounts for 60% of total metabolism [37]. Most brain energy is used in the maintenance and manipulation of ion gradients for synaptic transmissions and cortical development [34]. As the brain develops during the early neonatal period, certain connections are pruned and ATP must be spent for the creation of cell components [38].

To make up for increased metabolic requirements, clinicians need to respond appropriately by increasing nutritional availability to premature neonates. This can be a challenge because premature infants have immature digestive and absorptive capabilities [39]. Moreover, most immature infants must rely on total parenteral nutrition (TPN) for exogenous nutritional support [40]. TPN, however, can only safely provide a limited amount and concentration of nutrients. Furthermore, TPN use is associated with added potential complications which includes the following: (a) TPN may lead to a stunting of the neonatal intestinal development due to absence of trophic factors that are only released when nutrients are present in the intestinal lumen [41,42] and (b) Long-term use of TPN is associated with an increased risk of metabolic liver dysfunction [43]. Because of such concerns, premature neonates are given early trophic feedings, weaned off TPN gradually and are cautiously introduced to enteral nutrition through a nasogastric or an orogastric tube [42,44].

Despite these attempts to provide adequate nutrition, preterm infants tend to experience growth failure. Reali et al. found that in their cohort of premature infants born with a weight appropriate for gestational age (AGA, 2.5–4.0 kg), as many as 71.4% weighed less than the 10th percentile at discharge from the hospital [45]. It is currently unclear whether the cause of growth failure is due to inadequate nutrient supply to the infant or due to non-nutritional mechanisms that can also restrict growth and energy stores, such as inflammation or illness [2,3]. Nonetheless, it is clear that premature infants are frequently in an energy-deprived state due to increased metabolic demands combined with inadequate nutrition.

Under circumstances of frequent energy deprivation, premature infants compensate through tissue breakdown and protein loss [46]. Tissue and energy stores are converted into readily available fuel. Amino acids are recycled through the liver as carbon sources for gluconeogenesis or degraded into ketones, serving as the brain’s alternative fuel source [2]. As energy supply becomes increasingly scarce, tissue breakdown and energy storage utilization becomes necessary to provide fuel even for baseline cellular processes [47]. Under hypoxic conditions anaerobic metabolism is engaged, converting pyruvate to lactate and allowing the regeneration of nicotinamide-adenine dinucleotide (NAD^+^), so glycolysis can continue to generate ATP. Nonetheless, the resulting ATP is expended quickly, resulting in a steady decrease of ATP stores [23].

## 3. Energy Deficiency Affects Long-Term Outcomes

Energy deficits were shown to be detrimental to neurodevelopment and cognitive functions, especially in neonates [48]. The brain is a rapidly developing organ, which is responsible for 60% of total body’s energy requirements [37]. During development, a critical growth phase occurs, which refers to the period of time when the neonatal brain experiences a significant degree of neuroplasticity [49]. During this phase, the neonatal brain is highly influenced by nutritional availability [50]. Critical periods of growth are accompanied by an increase in metabolic demand, requiring adequate nutrition to support it. Stephens et. al identified the first week of life as a period of critical growth in extremely low birth weight infants (ELBW, infants who are born weighing less than 1000 g) and observed that increasing protein or energy intake during that time period correlated with improved neurodevelopmental outcomes at 18 months corrected age [51].

Despite the significant relationship between nutrition and growth and development, few tools exist that measure energy utilization and adequacy of nutrient intake in premature neonates. Current markers of nutritional status include growth velocity (15 g/k/day), weight gain (10–30 g/day) and head circumference (1 cm/week) [50,52]. However, these measures, are shown to only be applicable to a limited range of postnatal age [53]. For example, Fenton et al. [53] has shown that the commonly used weight growth velocity goal of 15 g/kg/d was only consistent with the preterm infant growth reference curves at about 34 weeks. Tools that measure the energy states of premature neonates throughout the wide range of gestational ages are needed. These tools need to be individualized to gestational age, birth weight, gender, and illness severity. For example, our laboratory examined urinary biochemical markers as a measure of ATP utilization in premature neonates [10,54]. In the late pre-term neonates, we found urinary hypoxanthine to be highest in those with respiratory disease, showing the effect of illness on ATP degradation [54]. This data suggests that neonates with respiratory disease may require higher total energy intake [53]. In addition, we found an association between urinary allantoin levels and the incidence of severe intraventricular hemorrhage (IVH) [55]. Allantoin is an oxidation product of uric acid in the presence of reactive oxygen species, which may be produced during increased ATP utilization [10]. Few studies on IVH and nutrition exist but Sammallahti et al. [56] observed that those with IVH had lower total energy intake and lower energy intake from human milk. This data suggests the importance of careful monitoring and provision of adequate nutrition in this vulnerable population. An additional challenge for neonatal growth assessment is that optimal growth is currently not well defined. Additionally, the few reported studies in neonatal growth are impacted by confounding factors that reduce the value of their conclusions [57]. As previously described, current premature infant growth models only take into account simple measurements such as length, weight, head circumference and body mass index (BMI) [58]. Furthermore, it is unclear if these premature infant growth models are appropriate because they are based on a population of healthier term infants [2]. In view of the absence of correlation between rapid growth of premature neonates with long-term adult metabolic risks and clear association with a decreased infant morbidity and mortality, improved nutritional support may be warranted [59].

In contrast to the established long-term neurodevelopmental outcomes of energy deficit, evidence regarding long-term metabolic and cardiovascular outcomes is unclear. Once a premature infant is stabilized in the NICU, nutrition is given and accelerated catch-up growth may occur within the first 24 months of postnatal life [60,61]. It is currently believed that catch-up growth is beneficial for the first 2 years of life [62], especially for neurodevelopment [3,50] but may have negative long-term consequences in adult metabolism [63]. Unfortunately, studies on the non-neurodevelopmental effects of catch-up growth are few and of poor quality [57]. Despite the benefits of catch-up growth, there are reports that preterm infants with accelerated weight gain after the first two years of life have higher relative body adiposity and cardiovascular complications compared to term infants [64,65,66]. In adults, such increased body adiposity is a risk factor for cardiovascular morbidity and metabolic syndrome [67]. Because of this, it was suggested that quality assessment of neonatal growth should include more comprehensive measures of body composition, such as fat mass (FM) vs. fat-free mass (FFM), instead of simple body length, weight, head circumference and BMI [58,68,69]. However, it remains unclear whether the increased FM in early postnatal life independently alters cardiovascular and metabolic health in adulthood.

Long-term effects of fetal growth, postnatal growth and early nutrition were studied with respect to cardiovascular and metabolic outcomes in preterm infants [70]. Adults, who were born prematurely, were found to have a significantly greater risk of developing hypertension and insulin resistance compared to those born at term. This difference, however, was not associated with body size, body composition, or FM distribution. Furthermore, growth between birth or expected term age and 12 to 18 months post-term, had no significant influence on blood pressure or metabolic syndrome in adulthood. Instead, it was suggested that growth during late infancy and childhood may have a more significant influence on later cardiovascular and metabolic health. This is consistent with a recent longitudinal cohort study [71] of association between weight gain in infancy and childhood with biomarkers of metabolic syndrome in adolescents who were born preterm. No significant correlation between infant weight gain and long-term metabolic consequences was observed, regardless of catch-up growth rate. Instead, significant associations were reported between childhood weight gain (after 1 year of age) and later body composition changes (*p* < 0.001 for higher fat mass, high fat index, lean mass index and waist circumference), higher fasting insulin (*p* = 0.002), lower insulin sensitivity (*p* < 0.001), higher systolic and diastolic blood pressures (*p* = 0.006 and 0.005, respectively), lower high density lipoprotein (HDL) (*p* = 0.001) and a higher total cholesterol to HDL ratio (*p* < 0.001). These studies support the novel idea that the growth velocity after the first two years of life is a more accurate predictor of adult risk of metabolic disorders than the catch-up growth rate of premature infants during the first two years of life [3,4].

## 4. Prematurity and Chronic Stress, Energy Deficiency and Neuroplasticity

Preterm infants are in a state of chronic physiological and biochemical stress due to prematurity, illness, medications and many unavoidable environmental stressors in the NICU. When the relationship between clinical handling procedures, stress, pain and energy expenditure was examined, it was observed that as the level of intervention increased, infant energy expenditure increased as well [72]. Additionally, a negative correlation was found between energy expenditure and oxygen saturation, supporting the hypothesis that oxygen desaturation events are likely to result in hypoxia, resulting in decreased ATP synthesis coupled with an increased ATP utilization. Our lab found a similar association of increased ATP utilization in response to TDPs [25].

Because preterm infants lack the agency to limit external stressful stimuli, they must rely on their caregivers to limit their exposure to stressors. These environmental stressors include loud sounds and alarms from clinical equipment, noise from other infants, handling by the caregivers themselves and constant interruption of sleep for medical procedures. Furthermore, premature infants are also exposed to multiple tissue damaging procedures (TDPs) for clinical care or diagnostic purposes [8,73]. These can have long-term consequences, including neuroplastic modulation of the neonates’ stress response.

The stress response can be represented by two concepts: allostasis and allostatic load [74]. Allostasis refers to the active process of metabolic or physiological adaptations in response to stressful stimuli. Allostatic load refers to the “wear and tear” of the body that increases over time in response to chronic stress [75]. Allostatic load can manifest itself as a dysregulation of the stress response due to a lack of adaptation, prolonged response, or inadequate response [75].

A key regulator of allostasis is the hypothalamic-pituitary-adrenal (HPA) axis. While the development and function of this axis still remains to be fully characterized in the newborn, stress regulation involves three main steps. First, the paraventricular nucleus of the hypothalamus synthesizes and secretes corticotrophin-releasing hormone (CRH). Second, the anterior lobe of the pituitary gland releases adrenocorticotropic hormone (ACTH) in response to CRH. Finally, cortisol is released by the adrenal glands in response to ACTH. For a healthy allostatic response, baseline hormone levels are restored through cortisol’s negative feedback loop to the hypothalamus and the pituitary gland. In the premature infant, the allostatic response via the HPA axis may be irreversibly altered due to chronic stress exposure. The allostatic load may be elevated due to insufficient cortisol production caused by illness or an underdeveloped HPA axis [76]. Heckmann et al. found that a mature adrenal response, defined in clinically stable premature infants as a tripled level of cortisol in response to stress, was present only in 27% (12 out of 44) of ill preterm infants [77]. However, these high responders were more prone to central nervous system (CNS) bleeds. Additionally, it was demonstrated that during the first 7 days of life, the pituitary gland is responsive to human CRH but cortisol production was suboptimal [78]. This may be due to an underdeveloped adrenal cortex or cortisol synthesis. This cortisol deficiency disappeared by day of life 14 [78]. Thus, allostasis may be inadequate in early life.

The HPA axis also plays a role in energy homeostasis. As part of the allostatic response, the metabolic demand for energy is augmented to increase chances of survival. One group [72] showed a significant correlation between stress and energy expenditure in premature neonates. Stress was defined as (1) a heart rate of less than 100 bpm or more than 160 bpm or an increased baseline of 5 bpm or more, (2) irregular respiratory rate of less than 40 or more than 60 breaths/min, or a baseline increase of 7 breaths/min or more and (3) oxygen saturation of less than 90% or a decrease of 2.5% or more. Energy expenditure was measured as follows:

$$E=\frac{M\times t}{H_{T}}$$

where *E* = energy expenditure per heartbeat $\left(\frac{calories}{\mathrm{kg}}\right)$, *M* = mean metabolic rate $\left(\frac{calories}{\mathrm{kg}\cdot \min}\right)$, *t* = duration of study (min) and *H_T_* = total accumulated heartbeats.

Stressful experiences may also have lasting developmental impact. Allostatic load can manifest itself through the dysregulation and neuroplastic modification of the HPA axis. Recently, an “ACTH-cortisol” dissociation was reported in critically ill adults, referring to low circulating ACTH coupled with elevated plasma cortisol [79,80]. Furthermore, high levels of chronic stress can alter HPA axis reactivity and shift the baseline set point of cortisol, blunting the allostatic response to acute stress [81]. A prolonged period is required to return to pre-stress hormone levels and higher concentrations of cortisol is required to respond to subsequent stressors [82]. Prolonged exposure to elevated cortisol levels may lead to increased proteolysis, which can negatively impact the overall growth of the neonate [83]. Chronic stress may also be associated with increased cognitive and behavioral problems and metabolic risks [84,85,86,87]. This can modify the structure and synaptic connections of the prefrontal cortex, the area of the brain associated with personality expression, decision-making and social behavior [88]. In rats, chronic stress was shown to increase synaptic inhibition of prefrontal glutamatergic output neurons, resulting in decreased control of stress reactivity and behavior [89]. Furthermore, chronic stress decreases synaptic density in the prefrontal cortex as well as in the hippocampus [90]. Thus, there are good reasons to expect, even in the absence of human newborn studies, that allostatic modifications in response to chronic stress may influence the neuronal development of the premature infant’s brain.

## 5. Sucrose and Stress Relief

Oral sucrose with non-nutritive sucking was shown in many studies to reduce procedural pain scores [11,12,13]. The evidence for the analgesic effects of sucrose is strongest for single event painful procedures such as heel lance, venipuncture, or intramuscular injection [7]. Analgesic benefits of sucrose for other painful procedures such as arterial puncture, subcutaneous injection, insertion of nasogastric or orogastric tubes, bladder catheterization, eye examinations or echocardiography examinations are less certain [7]. Most studies on sucrose examine pain behavior as the study variable. There is a limited amount of research on other outcome variables, such as cortisol. Some of these studies are outlined in Table 1.

A few studies in premature neonates measured the effects of sucrose administration on cortisol. Boyer et al. [91] found no significant difference in salivary cortisol concentration 30 min after a painful procedure in premature infants receiving either 24% sucrose or a placebo of sterile water. Stang et al. [92] found no significant difference in plasma cortisol 30 min after circumcision in groups receiving a dorsal penile nerve block agent and sucrose or water. Although sucrose had no significant effect on plasma cortisol levels, the pain scores decreased in both studies, which suggest that sucrose may only mask pain behavior with little effect on the glucocorticoid response [98]. A different response has been observed in animals. Ulrich-Lai and her colleagues showed that in rats exposed to chronic stress, sucrose consumption decreased corticotropin-releasing hormone messenger ribonucleic acid (CRH mRNA) in the paraventricular nucleus of the hypothalamus [96]. They also showed that the palatable and rewarding properties of sucrose are responsible for a decrease in adrenocorticotropic hormone (ACTH) and corticosterone [97]. The basolateral amygdala is altered in response to sucrose consumption and this alteration is long-lasting due to neuroplasticity [97]. Furthermore, increased duration and/or frequency of sucrose administration played a larger role in the dampening of the HPA axis than the volume of sucrose given, suggesting that sucrose may alter neuroplasticity and stress relief [99].

It was calculated that “*a 1000-gram infant receiving an average of 10 doses of 24% oral sucrose per day, at 0.5–1 mL per dose, is equal to a one year old infant receiving ½ can of regular Coke Classic per day*” [11]. The effect of this much sucrose on human premature infants is unknown. In adults, a 19-day study compared salivary cortisol levels between two groups that consumed either sucrose or aspartame along with a standardized, low-sugar baseline diet [93]. It was found that sucrose but not aspartame, reduced salivary cortisol levels after a comprehensive imaging stress test. In the same study, sucrose consumption correlated with a significant increase in activity levels in the left hippocampus, implying that sucrose inhibits stress induced deactivation of the hippocampus, perhaps through HPA axis suppression. These data suggest that oral sucrose consumption may modify the brain’s response to stress, specifically in the paraventricular nucleus of the hypothalamus and the basolateral amygdala. Interestingly, Stevens et al. showed that preterm infants <31 weeks’ gestational age who received >10 doses of sucrose per 24 h in the first week of life had poorer neurologic development compared with infants who received fewer sucrose doses [12]. However, in older premature neonates that are over 32 weeks gestational age, Banga et al. showed that repeated dosages of sucrose administration for procedural pain in premature infants for the first seven days after enrollment had no significant impact on neurobehavioral outcomes at 40 weeks post conception [94]. Additional studies are required to clarify the effect of sucrose analgesia on the newborn’s brain.

The mechanism for sucrose’s analgesic effect is unknown but is thought to be due to (a) the release of endogenous opioids two minutes after sucrose administration [100], although evidence to substantiate this hypothesis in humans is lacking, or (b) the occurrence of ingestion analgesia [101]. Ingestion analgesia occurs when hedonic foods are eaten and functions to defend eating from ending and stops when eating is over. Hedonic food is specific to the animal’s homeostatic state. For example, sodium becomes hedonic when effective circulating volume is low [102]. In a complementary manner, the sensation of thirst increases when plasma osmolality rises above normal levels [103]. Similarly, sucrose, an inherently hedonic food due to its sweet taste, has been shown to reduce stress via brain reward pathways [97]. Chronic stress modifies the brain’s reward pathway to increase the hedonic value of palatable high-calorie foods through the actions of glucocorticoids [16]. Incidentally, there is strong evidence that an animal’s energy stores may play a role in the regulation of the HPA axis [16]. The hedonic value of oral sucrose may be elevated in chronically stressed premature infants that are energy deficient, which may contribute to its effectiveness in decreasing the behavioral signs of pain.

Though sucrose may have a role in pain and stress relief, it may come at the cost of long-term neurologic and metabolic consequences and altered brain stress and reward pathways. Acutely, a single dose of oral sucrose administration for heel lance has been associated with increased ATP utilization and oxidative stress [10], perhaps due to the high metabolic cost of the fructose moiety of sucrose [104]. In a mouse pup model, the effects of early repeated sucrose treatment before an intervention on long-term brain structure was examined [95]. These mice pups received an oral dose of vehicle (sterile water) or 24% sucrose via a micropipette, two minutes before an intervention. The mice pups were separated into three different intervention groups: a needle-prick on the paw, light tactile paw pressure with a cotton swab, or only handling in a similar manner as the other groups. The mice pups received 10 interventions daily from post-natal day 1 (P1) to P6 to model the NICU experience. Adult brains were collected between P85 and P95 and were scanned using magnetic resonance imaging (MRI). Early repetitive sucrose exposure in mice resulted in smaller white matter volumes in the corpus callosum, stria terminalis and fimbria (*p* < 0.0001) and smaller cortical and subcortical gray matter in the hippocampus and cerebellum (*p* < 0.0001), regardless of intervention. This suggests that sucrose may affect brain development independent of procedural pain. The modulation of the HPA axis and the increased hedonic values placed on sweet solutions may also play a role in the increased risk of long-term negative metabolic outcomes. In the interim, however, there is not enough evidence to recommend the cessation of oral sucrose administration for procedural pain in the NICU. More studies are required to examine the analgesic effectiveness of metabolically “cheaper” sweet solutions, such as glucose, as well as other pharmacologic and non-pharmacologic methods to reduce pain.

## 6. Conclusions

In conclusion, premature infants are in a state of energy deficiency due to hypoxia, pain and stress, illness and neurodevelopment. Each of these factors increases ATP utilization, reducing energy stores. In addition, oral sucrose, a commonly used intervention for pain was recently shown to acutely increase ATP utilization as evidenced by increased biochemical markers of hypoxia and oxidative stress over time [10]. Nutritional support specific to a neonate’s age, weight, gender and illness severity needs to be provided to prevent energy deficit and tools that monitor energy states and efficacy of nutritional intake need to be developed and tested. Management of a neonate’s nutritional status is complex and requires prospective studies that will yield evidence-based methods and techniques.

## Figures and Tables

**Table 1:** Summary table of studies that examined other variables besides pain response after sucrose administration. (CNS, central nervous system; ACTH, adrenocorticotropic hormone; CRH, corticotropin-releasing hormone; mRNA, messenger ribonucleic acid)

Population	Parameter Measured	Effect	Sucrose Dose	Control	References
Premature Infant	ATP Utilization	Increased	2 mL for neonates >2 kg 1.5 mL for neonates 1.5–2 kg 0.5 mL for neonates that were <1.5 kg	Placebo (Sterile Water)	[10]
	Oxidative Stress	Increased			
Premature Infant	Salivary Cortisol	No Significant Difference	0.1–0.3 mL per Painful Procedure	Placebo (Sterile Water)	[91]
Premature Infant	Plasma Cortisol	No Significant Difference	Pacifier Dipped in Sucrose	Water	[92]
Adult Human Females	Salivary Cortisol	Decreased	3 Servings of Study Beverageper Day	Aspartame	[93]
	Regional Brain Responses	Increased in Left Hippocampus			
Premature Infant	Neurodevelopmental Assessment	No Significant Difference	0.5 mL per Painful Procedure	Placebo (Sterile Water)	[94]
Mouse Pups	CNS White Matter Regions	Decreased	0.1–0.2 g Sucrose per kg Body Weight	Vehicle (Sterile Water)	[95]
	CNS Gray Matter Regions	Decreased			
Adult Rat	Plasma ACTH	Decreased	Up to 4 mL Twice a Day	Saccharin and/or Water	[96,97]
	Corticosterone	Decreased			
	CRH mRNA	Decreased			

## References

[1] Dinerstein A., Nieto R.M., Solana C.L., Perez G.P., Otheguy L.E., Larguia A.M. (2006). Early and aggressive nutritional strategy (parenteral and enteral) decreases postnatal growth failure in very low birth weight infants. J. Perinatol..

[2] Ramel S.E., Brown L.D., Georgieff M.K. (2014). The Impact of Neonatal Illness on Nutritional Requirements—One Size Does Not Fit All. Curr. Pediatr. Rep..

[3] Ramel S.E., Demerath E.W., Gray H.L., Younge N., Boys C., Georgieff M.K. (2012). The relationship of poor linear growth velocity with neonatal illness and two-year neurodevelopment in preterm infants. Neonatology.

[4] Ehrenkranz R.A., Dusick A.M., Vohr B.R., Wright L.L., Wrage L.A., Poole W.K. (2006). Growth in the neonatal intensive care unit influences neurodevelopmental and growth outcomes of extremely low birth weight infants. Pediatrics.

[5] Lapillonne A. (2014). Feeding the preterm infant after discharge. World Rev. Nutr. Diet..

[6] Vargas J., Junco M., Gomez C., Lajud N. (2016). Early Life Stress Increases Metabolic Risk, HPA Axis Reactivity and Depressive-Like Behavior When Combined with Postweaning Social Isolation in Rats. PLoS ONE.

[7] Stevens B., Yamada J., Ohlsson A., Haliburton S., Shorkey A. (2016). Sucrose for analgesia (pain relief) in newborn infants undergoing painful procedures. Cochrane Database Syst. Rev..

[8] Carbajal R., Rousset A., Danan C., Coquery S., Nolent P., Ducrocq S., Saizou C., Lapillonne A., Granier M., Durand P. (2008). Epidemiology and treatment of painful procedures in neonates in intensive care units. JAMA.

[9] Angeles D.M., Ashwal S., Wycliffe N.D., Ebner C., Fayard E., Sowers L., Holshouser B.A. (2007). Relationship between opioid therapy, tissue-damaging procedures and brain metabolites as measured by proton MRS in asphyxiated term neonates. Pediatr. Res..

[10] Asmerom Y., Slater L., Boskovic D.S., Bahjri K., Holden M.S., Phillips R., Deming D., Ashwal S., Fayard E., Angeles D.M. (2013). Oral sucrose for heel lance increases adenosine triphosphate use and oxidative stress in preterm neonates. J. Pediatr..

[11] Holsti L., Grunau R.E. (2010). Considerations for using sucrose to reduce procedural pain in preterm infants. Pediatrics.

[12] Stevens B., Yamada J., Beyene J., Gibbins S., Petryshen P., Stinson J., Narciso J. (2005). Consistent management of repeated procedural pain with sucrose in preterm neonates: Is it effective and safe for repeated use over time?. Clin. J. Pain.

[13] Taddio A., Shah V., Atenafu E., Katz J. (2009). Influence of repeated painful procedures and sucrose analgesia on the development of hyperalgesia in newborn infants. Pain.

[14] Muralidhara D.V., Shetty P.S. (1990). Sucrose feeding stimulates basal metabolism & nonshivering thermogenesis in undernourished rats. Indian J. Med. Res..

[15] Laugero K.D. (2001). A new perspective on glucocorticoid feedback: Relation to stress, carbohydrate feeding and feeling better. J. Neuroendocrinol..

[16] Laugero K.D. (2004). Reinterpretation of basal glucocorticoid feedback: Implications to behavioral and metabolic disease. Vitam. Horm..

[17] Goran M.I., Dumke K., Bouret S.G., Kayser B., Walker R.W., Blumberg B. (2013). The obesogenic effect of high fructose exposure during early development. Nat. Rev. Endocrinol..

[18] Tappy L., Egli L., Lecoultre V., Schneider P. (2013). Effects of fructose-containing caloric sweeteners on resting energy expenditure and energy efficiency: A review of human trials. Nutr. Metab..

[19] Goldenberg R.L., Culhane J.F., Iams J.D., Romero R. (2008). Epidemiology and causes of preterm birth. Lancet.

[20] Tchirikov M., Zhumadilov Z.S., Bapayeva G., Bergner M., Entezami M. (2017). The effect of intraumbilical fetal nutrition via a subcutaneously implanted port system on amino acid concentration by severe IUGR human fetuses. J. Perinat. Med..

[21] Denne S.C. (2001). Protein and energy requirements in preterm infants. Semin. Neonatol. SN.

[22] Fairchild K., Mohr M., Paget-Brown A., Tabacaru C., Lake D., Delos J., Moorman J.R., Kattwinkel J. (2016). Clinical associations of immature breathing in preterm infants: Part 1—Central apnea. Pediatr. Res..

[23] Plank M.S., Boskovic D.S., Sowers L.C., Angeles D.M. (2008). Biochemical markers of neonatal hypoxia. Pediatr. Health.

[24] Michiels C. (2004). Physiological and pathological responses to hypoxia. Am. J. Pathol..

[25] Slater L., Asmerom Y., Boskovic D.S., Bahjri K., Plank M.S., Angeles K.R., Phillips R., Deming D., Ashwal S., Hougland K. (2012). Procedural pain and oxidative stress in premature neonates. J. Pain Off. J. Am. Pain Soc..

[26] Holsti L., Grunau R.E., Oberlander T.F., Whitfield M.F., Weinberg J. (2005). Body movements: An important additional factor in discriminating pain from stress in preterm infants. Clin. J. Pain.

[27] Flatters S.J. (2015). The Contribution of Mitochondria to Sensory Processing and Pain. Prog. Mol. Biol. Transl. Sci..

[28] Steinhorn D.M., Green T.P. (1991). Severity of illness correlates with alterations in energy metabolism in the pediatric intensive care unit. Crit. Care Med..

[29] Cerra F.B., Siegel J.H., Coleman B., Border J.R., McMenamy R.R. (1980). Septic autocannibalism. A failure of exogenous nutritional support. Ann. Surg..

[30] Mehta N.M., Duggan C.P. (2009). Nutritional Deficiencies during Critical Illness. Pediatr. Clin. N. Am..

[31] Dao D.T., Anez-Bustillos L., Cho B.S., Li Z., Puder M., Gura K.M. (2017). Assessment of Micronutrient Status in Critically Ill Children: Challenges and Opportunities. Nutrients.

[32] De Albuquerque Wilasco M.I., Uribe-Cruz C., Santetti D., Fries G.R., Dornelles C.T.L., da Silveira T.R. (2017). IL-6, TNF-α, IL-10 and nutritional status in pediatric patients with biliary atresia. J. Pediatr. (Rio J.).

[33] Harris M.C., Costarino A.T., Sullivan J.S., Dulkerian S., McCawley L., Corcoran L., Butler S., Kilpatrick L. (1994). Cytokine elevations in critically ill infants with sepsis and necrotizing enterocolitis. J. Pediatr..

[34] Harris J.J., Jolivet R., Attwell D. (2012). Synaptic energy use and supply. Neuron.

[35] Brummelte S., Grunau R.E., Chau V., Poskitt K.J., Brant R., Vinall J., Gover A., Synnes A.R., Miller S.P. (2012). Procedural pain and brain development in premature newborns. Ann. Neurol..

[36] Miller S.P., Ferriero D.M. (2009). From selective vulnerability to connectivity: Insights from newborn brain imaging. Trends Neurosci..

[37] Kuzawa C.W. (1998). Adipose tissue in human infancy and childhood: An evolutionary perspective. Am. J. Phys. Anthropol..

[38] Harris J.J., Reynell C., Attwell D. (2011). The physiology of developmental changes in BOLD functional imaging signals. Dev. Cogn. Neurosci..

[39] Hay W.W., Brown L.D., Denne S.C. (2014). Energy requirements, protein-energy metabolism and balance and carbohydrates in preterm infants. World Rev. Nutr. Diet..

[40] Neu J. (2007). Gastrointestinal development and meeting the nutritional needs of premature infants. Am. J. Clin. Nutr..

[41] Burrin D.G., Stoll B. (2002). Key nutrients and growth factors for the neonatal gastrointestinal tract. Clin. Perinatol..

[42] Jacobi S.K., Odle J. (2012). Nutritional Factors Influencing Intestinal Health of the Neonate. Adv. Nutr. Int. Rev. J..

[43] Stoll B., Horst D.A., Cui L., Chang X., Ellis K.J., Hadsell D.L., Suryawan A., Kurundkar A., Maheshwari A., Davis T.A. (2010). Chronic Parenteral Nutrition Induces Hepatic Inflammation, Steatosis and Insulin Resistance in Neonatal Pigs. J. Nutr..

[44] Tappenden K.A. (2006). Mechanisms of enteral nutrient-enhanced intestinal adaptation. Gastroenterology.

[45] Reali A., Greco F., Marongiu G., Deidda F., Atzeni S., Campus R., Dessì A., Fanos V. (2015). Individualized fortification of breast milk in 41 Extremely Low Birth Weight (ELBW) preterm infants. Clin. Chim. Acta.

[46] Ibrahim H.M., Jeroudi M.A., Baier R.J., Dhanireddy R., Krouskop R.W. (2004). Aggressive early total parental nutrition in low-birth-weight infants. J. Perinatol. Off. J. Calif. Perinat. Assoc..

[47] Shalak L., Perlman J.M. (2004). Hypoxic-ischemic brain injury in the term infant-current concepts. Early Hum. Dev..

[48] Georgieff M.K., Brunette K.E., Tran P.V. (2015). Early life nutrition and neural plasticity. Dev. Psychopathol..

[49] Hensch T.K. (2004). Critical period regulation. Annu. Rev. Neurosci..

[50] Ramel S.E., Georgieff M.K. (2014). Preterm nutrition and the brain. World Rev. Nutr. Diet..

[51] Stephens B.E., Walden R.V., Gargus R.A., Tucker R., McKinley L., Mance M., Nye J., Vohr B.R. (2009). First-week protein and energy intakes are associated with 18-month developmental outcomes in extremely low birth weight infants. Pediatrics.

[52] Isaacs E.B., Morley R., Lucas A. (2009). Early diet and general cognitive outcome at adolescence in children born at or below 30 weeks gestation. J. Pediatr..

[53] Fenton T.R., Anderson D., Groh-Wargo S., Hoyos A., Ehrenkranz R.A., Senterre T. (2017). An Attempt to Standardize the Calculation of Growth Velocity of Preterm Infants-Evaluation of Practical Bedside Methods. J. Pediatr..

[54] Holden M.S., Hopper A., Slater L., Asmerom Y., Esiaba I., Boskovic D.S., Angeles D.M. (2014). Urinary Hypoxanthine as a Measure of Increased ATP Utilization in Late Preterm Infants. ICAN Infant Child Adolesc. Nutr..

[55] Esiaba I., Angeles D.M., Holden M.S., Tan J.B.C., Asmerom Y., Gollin G., Boskovic D.S. (2016). Urinary Allantoin Is Elevated in Severe Intraventricular Hemorrhage in the Preterm Newborn. Transl. Stroke Res..

[56] Sammallahti S., Kajantie E., Matinolli H.-M., Pyhälä R., Lahti J., Heinonen K., Lahti M., Pesonen A.-K., Eriksson J.G., Hovi P. (2017). Nutrition after preterm birth and adult neurocognitive outcomes. PLoS ONE.

[57] Martin A., Connelly A., Bland R.M., Reilly J.J. (2016). Health impact of catch-up growth in low-birth weight infants: Systematic review, evidence appraisal and meta-analysis. Matern. Child. Nutr..

[58] Rice M.S., Valentine C.J. (2015). Neonatal Body Composition: Measuring Lean Mass as a Tool to Guide Nutrition Management in the Neonate. Nutr. Clin. Pract. Off. Publ. Am. Soc. Parenter. Enter. Nutr..

[59] Raaijmakers A., Allegaert K. (2016). Catch-Up Growth in Former Preterm Neonates: No Time to Waste. Nutrients.

[60] Jaquet D., Deghmoun S., Chevenne D., Collin D., Czernichow P., Lévy-Marchal C. (2005). Dynamic change in adiposity from fetal to postnatal life is involved in the metabolic syndrome associated with reduced fetal growth. Diabetologia.

[61] Hay W.W. (2013). Aggressive Nutrition of the Preterm Infant. Curr. Pediatr. Rep..

[62] Victora C.G., Barros F.C., Horta B.L., Martorell R. (2001). Short-term benefits of catch-up growth for small-for-gestational-age infants. Int. J. Epidemiol..

[63] Jain V., Singhal A. (2012). Catch up growth in low birth weight infants: Striking a healthy balance. Rev. Endocr. Metab. Disord..

[64] Ramel S.E., Gray H.L., Ode K.L., Younge N., Georgieff M.K., Demerath E.W. (2011). Body composition changes in preterm infants following hospital discharge: Comparison with term infants. J. Pediatr. Gastroenterol. Nutr..

[65] Olhager E., Törnqvist C. (2014). Body composition in late preterm infants in the first 10 days of life and at full term. Acta Paediatr..

[66] Johnson M.J., Wootton S.A., Leaf A.A., Jackson A.A. (2012). Preterm birth and body composition at term equivalent age: A systematic review and meta-analysis. Pediatrics.

[67] Franco L.P., Morais C.C., Cominetti C. (2016). Normal-weight obesity syndrome: Diagnosis, prevalence and clinical implications. Nutr. Rev..

[68] Rigo J., de Curtis M., Pieltain C. (2001). Nutritional assessment in preterm infants with special reference to body composition. Semin. Neonatol..

[69] Griffin I.J. (2007). Nutritional assessment in preterm infants. Nestlé Nutr. Workshop Ser. Paediatr. Programme.

[70] Lapillonne A., Griffin I.J. (2013). Feeding preterm infants today for later metabolic and cardiovascular outcomes. J. Pediatr..

[71] Embleton N.D., Korada M., Wood C.L., Pearce M.S., Swamy R., Cheetham T.D. (2016). Catch-up growth and metabolic outcomes in adolescents born preterm. Arch. Dis. Child..

[72] Peng N.-H., Bachman J., Chen C.-H., Huang L.-C., Lin H.-C., Li T.-C. (2014). Energy expenditure in preterm infants during periods of environmental stress in the neonatal intensive care unit. Jpn. J. Nurs. Sci. JJNS.

[73] Stevens B., Yamada J., Lee G.Y., Ohlsson A. (2013). Sucrose for analgesia in newborn infants undergoing painful procedures. Cochrane Database Syst. Rev..

[74] Atkinson L., Jamieson B., Khoury J., Ludmer J., Gonzalez A. (2016). Stress Physiology in Infancy and Early Childhood: Cortisol Flexibility, Attunement and Coordination. J. Neuroendocrinol..

[75] McEwen B.S. (1998). Stress, adaptation and disease. Allostasis and allostatic load. Ann. N. Y. Acad. Sci..

[76] Fernandez E.F., Watterberg K.L. (2009). Relative adrenal insufficiency in the preterm and term infant. J. Perinatol. Off. J. Calif. Perinat. Assoc..

[77] Heckmann M., Hartmann M.F., Kampschulte B., Gack H., Bödeker R.-H., Gortner L., Wudy S.A. (2005). Cortisol production rates in preterm infants in relation to growth and illness: A noninvasive prospective study using gas chromatography-mass spectrometry. J. Clin. Endocrinol. Metab..

[78] Ng P.C. (2011). Effect of stress on the hypothalamic-pituitary-adrenal axis in the fetus and newborn. J. Pediatr..

[79] Boonen E., Berghe G.V. (2014). Novel insights in the HPA-axis during critical illness. Acta Clin. Belg..

[80] Peeters B., Boonen E., Langouche L., Van den Berghe G. (2015). The HPA axis response to critical illness: New study results with diagnostic and therapeutic implications. Mol. Cell. Endocrinol..

[81] Stephens M.A.C., Wand G. (2012). Stress and the HPA Axis. Alcohol Res. Curr. Rev..

[82] McEwen B.S., Gianaros P.J. (2010). Central role of the brain in stress and adaptation: Links to socioeconomic status, health and disease. Ann. N. Y. Acad. Sci..

[83] Simmons P.S., Miles J.M., Gerich J.E., Haymond M.W. (1984). Increased proteolysis. An effect of increases in plasma cortisol within the physiologic range. J. Clin. Investig..

[84] Haley D.W., Weinberg J., Grunau R.E. (2006). Cortisol, contingency learning and memory in preterm and full-term infants. Psychoneuroendocrinology.

[85] Quesada A.A., Tristão R.M., Pratesi R., Wolf O.T. (2014). Hyper-responsiveness to acute stress, emotional problems and poorer memory in former preterm children. Stress Amst. Neth..

[86] Wadsby M., Nelson N., Ingemansson F., Samuelsson S., Leijon I. (2014). Behaviour problems and cortisol levels in very-low-birth-weight children. Nord. J. Psychiatry.

[87] Juruena M.F. (2014). Early-life stress and HPA axis trigger recurrent adulthood depression. Epilepsy Behav..

[88] Yang Y., Raine A. (2009). Prefrontal Structural and Functional Brain Imaging findings in Antisocial, Violent and Psychopathic Individuals: A Meta-Analysis. Psychiatry Res..

[89] McKlveen J.M., Morano R.L., Fitzgerald M., Zoubovsky S., Cassella S.N., Scheimann J.R., Ghosal S., Mahbod P., Packard B.A., Myers B. (2016). Chronic Stress Increases Prefrontal Inhibition: A Mechanism for Stress-Induced Prefrontal Dysfunction. Biol. Psychiatry.

[90] Reser J.E. (2016). Chronic stress, cortical plasticity and neuroecology. Behav. Processes.

[91] Boyer K., Johnston C., Walker C.-D., Filion F., Sherrard A. (2004). Does sucrose analgesia promote physiologic stability in preterm neonates?. Biol. Neonate.

[92] Stang H.J., Snellman L.W., Condon L.M., Conroy M.M., Liebo R., Brodersen L., Gunnar M.R. (1997). Beyond dorsal penile nerve block: A more humane circumcision. Pediatrics.

[93] Tryon M.S., Stanhope K.L., Epel E.S., Mason A.E., Brown R., Medici V., Havel P.J., Laugero K.D. (2015). Excessive Sugar Consumption May Be a Difficult Habit to Break: A View From the Brain and Body. J. Clin. Endocrinol. Metab..

[94] Banga S., Datta V., Rehan H.S., Bhakhri B.K. (2016). Effect of Sucrose Analgesia, for Repeated Painful Procedures, on Short-term Neurobehavioral Outcome of Preterm Neonates: A Randomized Controlled Trial. J. Trop. Pediatr..

[95] Tremblay S., Ranger M., Chau C.M.Y., Ellegood J., Lerch J.P., Holsti L., Goldowitz D., Grunau R.E. (2017). Repeated exposure to sucrose for procedural pain in mouse pups leads to long-term widespread brain alterations. Pain.

[96] Ulrich-Lai Y.M., Ostrander M.M., Thomas I.M., Packard B.A., Furay A.R., Dolgas C.M., Van Hooren D.C., Figueiredo H.F., Mueller N.K., Choi D.C. (2007). Daily limited access to sweetened drink attenuates hypothalamic-pituitary-adrenocortical axis stress responses. Endocrinology.

[97] Ulrich-Lai Y.M., Christiansen A.M., Ostrander M.M., Jones A.A., Jones K.R., Choi D.C., Krause E.G., Evanson N.K., Furay A.R., Davis J.F. (2010). Pleasurable behaviors reduce stress via brain reward pathways. Proc. Natl. Acad. Sci. USA.

[98] Slater R., Cornelissen L., Fabrizi L., Patten D., Yoxen J., Worley A., Boyd S., Meek J., Fitzgerald M. (2010). Oral sucrose as an analgesic drug for procedural pain in newborn infants: A randomised controlled trial. Lancet.

[99] Ulrich-Lai Y.M., Ostrander M.M., Herman J.P. (2011). HPA axis dampening by limited sucrose intake: Reward frequency vs. caloric consumption. Physiol. Behav..

[100] Blass E.M., Ciaramitaro V. (1994). A new look at some old mechanisms in human newborns: Taste and tactile determinants of state, affect and action. Monogr. Soc. Res. Child Dev..

[101] Fitzgerald M. (2015). What do we really know about newborn infant pain?. Exp. Physiol..

[102] Foo H., Mason P. (2011). Ingestion analgesia occurs when a bad taste turns good. Behav. Neurosci..

[103] Takamata A., Mack G.W., Gillen C.M., Nadel E.R. (1994). Sodium appetite, thirst and body fluid regulation in humans during rehydration without sodium replacement. Am. J. Physiol..

[104] Tappy L., Lê K.-A. (2010). Metabolic effects of fructose and the worldwide increase in obesity. Physiol. Rev..

